# Gene Expression in Chicken Reveals Correlation with Structural Genomic Features and Conserved Patterns of Transcription in the Terrestrial Vertebrates

**DOI:** 10.1371/journal.pone.0011990

**Published:** 2010-08-05

**Authors:** Haisheng Nie, Richard P. M. A. Crooijmans, Aart Lammers, Evert M. van Schothorst, Jaap Keijer, Pieter B. T. Neerincx, Jack A. M. Leunissen, Hendrik-Jan Megens, Martien A. M. Groenen

**Affiliations:** 1 Animal Breeding and Genomics Centre, Wageningen University, Wageningen, The Netherlands; 2 Adaptation Physiology Group, Wageningen University, Wageningen, The Netherlands; 3 Human and Animal Physiology, Wageningen University, Wageningen, The Netherlands; 4 Laboratory of Bioinformatics, Wageningen University, Wageningen, The Netherlands; Institute of Infectious Disease and Molecular Medicine, South Africa

## Abstract

**Background:**

The chicken is an important agricultural and avian-model species. A survey of gene expression in a range of different tissues will provide a benchmark for understanding expression levels under normal physiological conditions in birds. With expression data for birds being very scant, this benchmark is of particular interest for comparative expression analysis among various terrestrial vertebrates.

**Methodology/Principal Findings:**

We carried out a gene expression survey in eight major chicken tissues using whole genome microarrays. A global picture of gene expression is presented for the eight tissues, and tissue specific as well as common gene expression were identified. A Gene Ontology (GO) term enrichment analysis showed that tissue-specific genes are enriched with GO terms reflecting the physiological functions of the specific tissue, and housekeeping genes are enriched with GO terms related to essential biological functions. Comparisons of structural genomic features between tissue-specific genes and housekeeping genes show that housekeeping genes are more compact. Specifically, coding sequence and particularly introns are shorter than genes that display more variation in expression between tissues, and in addition intergenic space was also shorter. Meanwhile, housekeeping genes are more likely to co-localize with other abundantly or highly expressed genes on the same chromosomal regions. Furthermore, comparisons of gene expression in a panel of five common tissues between birds, mammals and amphibians showed that the expression patterns across tissues are highly similar for orthologuous genes compared to random gene pairs within each pair-wise comparison, indicating a high degree of functional conservation in gene expression among terrestrial vertebrates.

**Conclusions:**

The housekeeping genes identified in this study have shorter gene length, shorter coding sequence length, shorter introns, and shorter intergenic regions, there seems to be selection pressure on economy in genes with a wide tissue distribution, i.e. these genes are more compact. A comparative analysis showed that the expression patterns of orthologous genes are conserved in the terrestrial vertebrates during evolution.

## Introduction

The chicken is an important model species for evolutionary and developmental biology, immunology, genetics, as well as for agricultural science. The completion of a draft sequence of the chicken genome [1] represented a landmark in avian genomics and has opened new possibilities to understand gene function and its relationship to physiology. Often gene functions of chicken genes were annotated based on sequence conservation without further functional evidence. A survey of gene expression in a range of different tissues under normal physiological conditions, therefore, would provide additional support for the potential function of many of the chicken genes.

Several studies, using chicken as a model, have compared gene expression differences under different infection treatments using microarrays [2]–[6]. Most of these studies surveyed gene expression in a single tissue (mostly immune related) and identified genes differentially expressed between two or more conditions (control vs. treatments) in the tissue of interest. However, the identified marker genes for diagnosis and molecular targets for vaccines will depend on knowledge not only of the genes expressed in the diseased tissues of interest, but also on detailed information about the expression of the corresponding genes across different normal tissues. In chicken, the global expression pattern of the genes under normal physiological conditions across a range of tissues and developmental stages needs to be surveyed to provide a global picture of the chicken transcriptome. This information would provide a baseline for future expression studies on diseases and other traits in chickens, and understanding global distribution of gene expression among several tissues would aid in identifying genes with housekeeping functions and genes with tissue-specific functions. In humans housekeeping genes were found to have relatively shorter introns, untranslated regions and coding sequences, suggesting a selection for compactness of genes that show a wide tissue distribution of expression [7], [8]. While this phenomenon is thought to be universally present in all vertebrates, it has been corroborated by a limited number of studies so far. One of the aims of the current study is to establish the relationship between gene compactness and specificity in expression in birds.

Furthermore, clustering of highly expressed genes within specific chromosomal regions has been reported in human [9], mouse [10], chicken [11], and fruit fly [12]. These regions were termed “RIDGEs” (Regions of Increased Gene Expression). RIDGEs were reported to be associated with higher expression, higher gene density, shorter gene introns, shorter genes, and some other genomic features in chicken [11]. Shorter introns were also reported for highly expressed genes in the human genome [13], and the authors hypothesized that transcription efficiency is enhanced when intron length is shorter. In the current study, we present the analysis of gene expression data and investigate the relationship between chromosomal locations and widely expressed genes in chicken.

Evolutionary changes in gene expression account for most phenotypic differences between different species. Studies on conservation of global gene expression patterns between human and apes [14], human and mouse [15] and different other vertebrate species [16] have been reported previously. The results of these studies suggested that the gene expressions within mammals and even within vertebrates are globally conserved, but corroborations of this phenomenon including the largest group of terrestrial vertebrates – i.e. the birds – so far has been scant. Here we present a comparative analysis on gene expression in three phylogenetically disparate clades in the terrestrial vertebrates: birds, mammals, and amphibians. Using this comparative approach, we tested whether the conservation of gene expression is correlated with species divergence time.

To summarize, the objectives of this study are to address the following questions: 1) what is the distribution of gene expression in chicken? 2) Do genes with distinct breadth of expression (number of tissues where a gene is expressed) show a correlation with certain structural genomic features in chicken? 3) Are the expression patterns of orthologous genes conserved among species?

## Results

### Gene expression distribution in different chicken tissues

**Figure 1:**
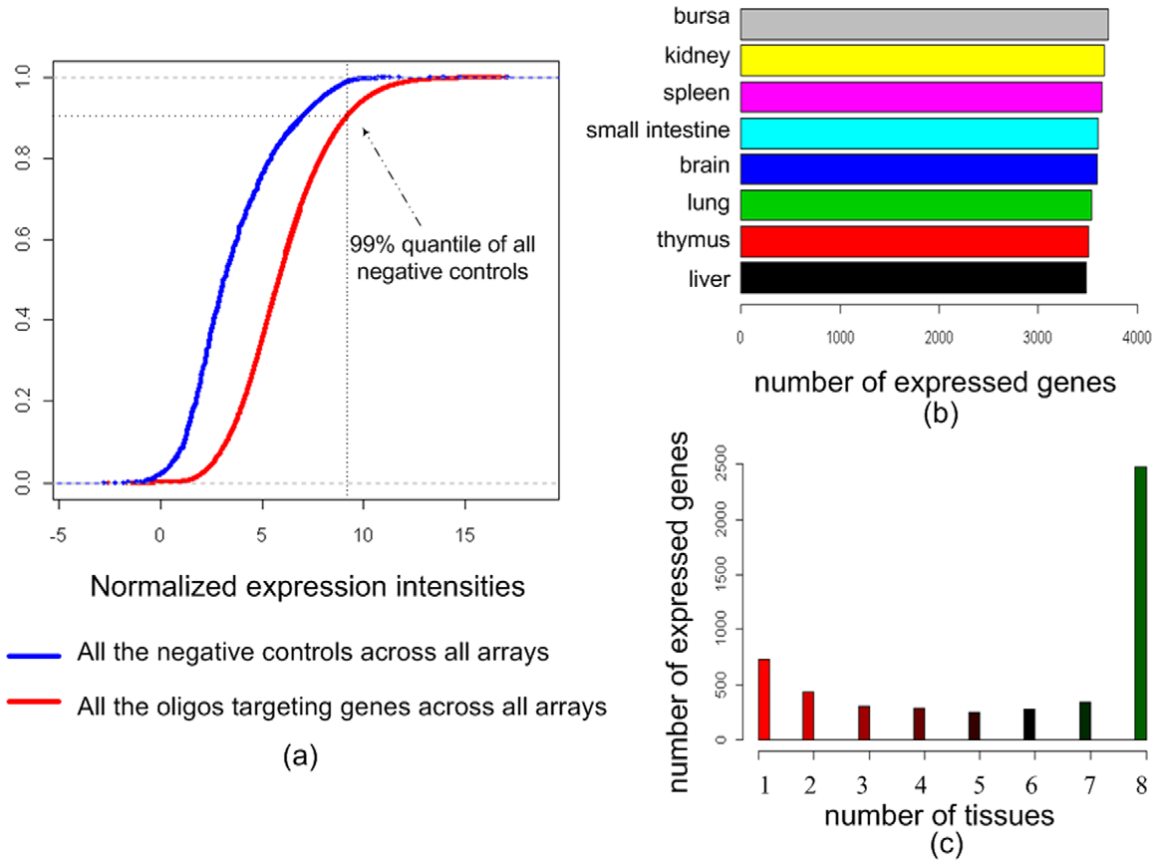
Summary of chicken gene expression data: (a). Accumulative plots of arcsihn transformed intensity of genes and negative controls on all the arrays, the red line in Figure 1a indicates all the gene probes on the array and the blue line indicates all the negative control spots across all the arrays. (b). Number of genes expressed in eight chicken tissues (c) Distribution of number of tissues in which genes are expressed (for example, 1 represents the tissue-specific genes, i.e. genes only expressed in one individual tissues, 2 represents that genes are expressed in two tissues out of the eight, and so on, 8 represents that genes are expressed in all eight tissues.)

**Figure 2:**
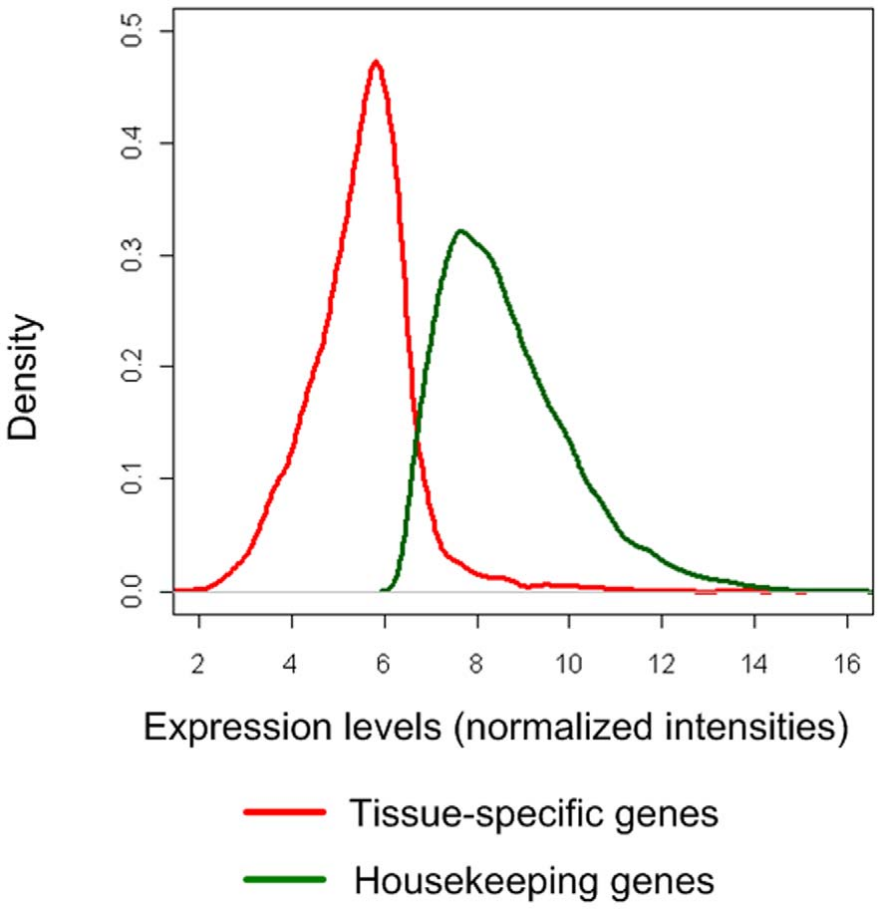
Density plot of expression levels for tissue-specific genes (red line) and housekeeping genes (green line) across eight chicken tissues.

Normalized intensities were used as gene expression levels and genes were defined as being expressed only when their expression was higher than 99% quantile value of the expression of all negative control spots across all the arrays in this study (Figure 1a) as described by Zhang et al. [17]. The probe annotations were updated by mapping the probe sequences to the current chicken genome assembly (WASHUC 2, May 2006) using the approach as described by Neerincx et al. [18]. In total, 14,900 probes out of the 20460 probes were mapped uniquely to the chicken assembly, representing 8,908 unique genes (8,792 Ensembl genes and 116 Entrez genes). The expression data for these genes is available in Table S1. Overall, 57% of the genes are expressed in at least one of the eight tissues (5,086 out of total 8,908 genes represented on the array platform (see materials and methods)). The number of genes expressed in each of the eight individual tissues was similar (Figure 1b) with on average, about 40% of the genes being expressed in each individual tissue type. The distribution of gene expression (number of tissues where a gene is expressed) is shown in Figure 1c. In total, 723 genes showed a single-tissue-specific pattern of expression, whereas 2,476 genes were found to be expressed in all eight tissues (Table S2). In this study, we refer to these 723 genes expressed only in one individual tissue as “tissue-specific genes”, and to the 2,476 genes expressed in all eight tissues as “housekeeping genes”. The expression levels of housekeeping genes across eight tissues were higher compared to tissue-specific genes (Figure 2). A GO terms enrichment analysis was performed using GOstats [19] on tissue-specific genes in each tissue type and on the housekeeping genes. The significant (p value<0.01) GO terms for Biological Process (BP) of the tissue-specific genes are shown in Table S3. The GO terms enriched for each tissue-specific gene list nicely correlates with the physiological function of the individual organs. For example, brain specific genes have enriched GO terms like “neurogenesis”, “nervous system development”, “neurotransmitter secretion”, and “learning” while liver specific genes have enriched GO terms like “blood coagulation”, “response to wounding” and “positive regulation of angiogenesis”, functions one typically might expect from brain and liver tissues, respectively.

The significant (p value<0.01) GO terms (BP) of housekeeping genes indicate that these widely expressed genes are mainly involved in a number of essential biological processes for maintaining a cell (Table S4). GO terms like “translation”, “protein folding”, “protein localization”, “rRNA processing” and “regulation of gene expression” indicate that most of these housekeeping genes are involved in regulation of transcription and translation.

### Housekeeping genes are compact compared to tissue-specific genes

**Figure 3:**
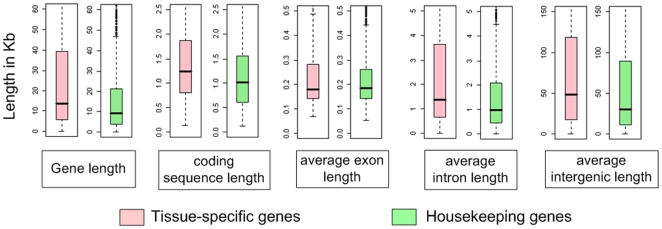
Box plot of genomic features for tissue-specific genes and housekeeping genes identified based on gene expression in eight chicken tissues: gene length, cds length, average exon length, average intron length, and intergenic length.

Besides the distinct functions of housekeeping genes compared to tissue-specific genes, we also examined the genomic features, e.g. gene length, coding sequence length, average exon length, average intron length, and intergenic region length, of both the 2,476 housekeeping genes and the 723 tissue-specific genes. Significant differences of gene length (p value = 1.4×10^−13^, Wilcoxon Rank Sum Test), coding sequence length (p value = 3.1×10^−13^), average intron length (p value = 3.7×10^−13^), and intergenic region length (p value = 5.8×10^−9^) were found between housekeeping and tissue-specific genes, housekeeping genes have, on average, shorter average exon length than tissue-specific genes, but the difference is not statistically significant (p value = 0.96) (Figure 3). These results suggest that in chicken housekeeping genes are relatively more compact than tissue-specific genes.

### Chicken housekeeping genes are significantly more located in RIDGEs comparing to random situations

A chicken transcriptome map is described previously, and regions with clusters of the most highly expressed genes, covering about 10% of the chicken genome, so called “RIDGEs”, are identified [11]. We checked the genomic locations of all 2,476 housekeeping genes in this study and found that about 31% (741 genes) of the housekeeping genes are located within RIDGEs in the chicken genome. To test the significance of the favorable distribution of housekeeping genes within RIDGEs, we performed a random permutation analysis by sampling 2,476 random genes for 1000 times from all 8,908 genes included in this analysis and computed the percentages of random genes being located within RIDGEs. Compared to housekeeping genes, randomly selected genes are much less often located in RIDGEs (13±0.6%, mean±sd). Therefore, the genomic locations of house-keeping genes show a higher overlap with RIDGEs across the chicken genome.

### Expression of orthologous genes is conserved in vertebrates

**Figure 4:**
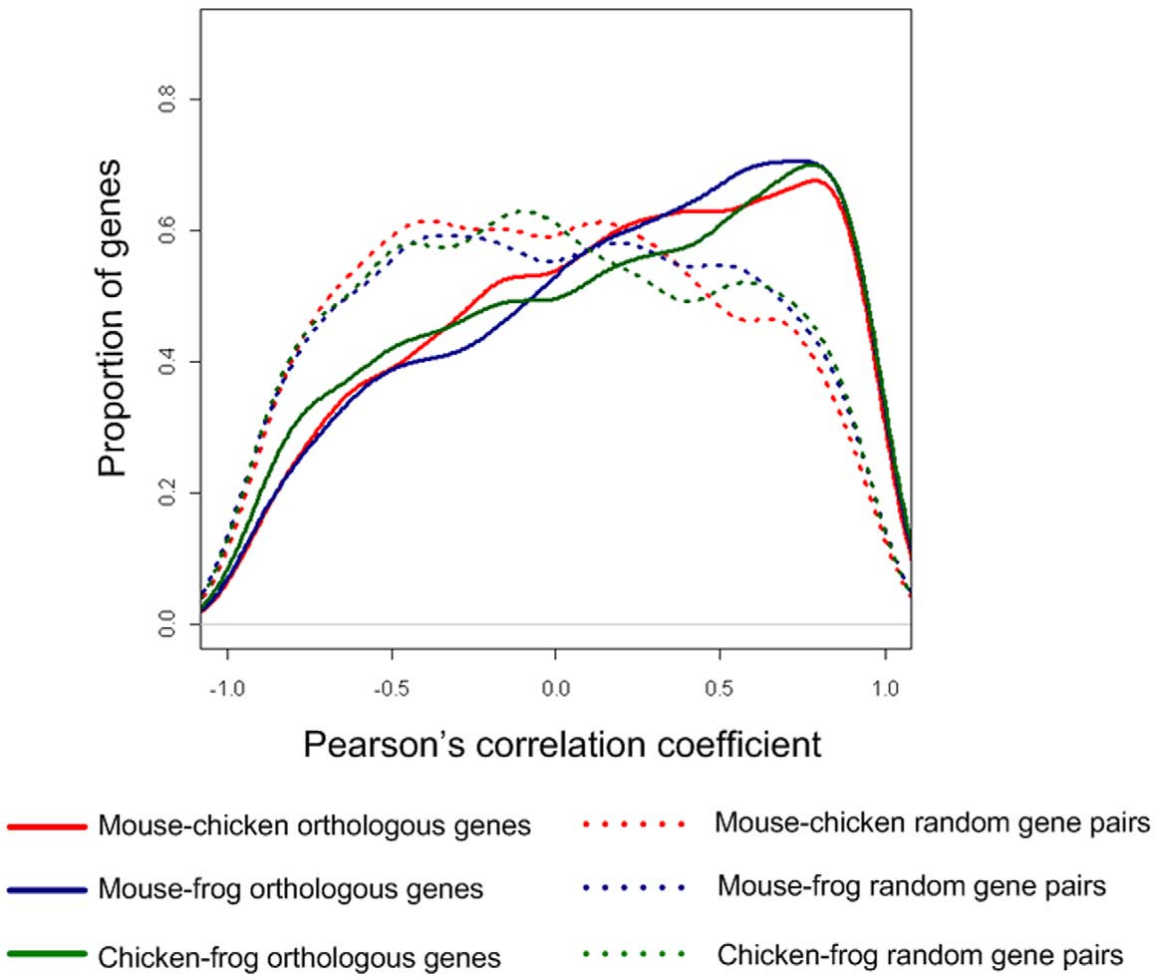
Distribution of gene expression correlation coefficients of orthologous gene pairs and random gene pairs in pair-wise comparisons among mouse, chicken, and frog.

Conservation of gene expression was compared by checking the 3,892 1∶1∶1 orthologous genes in mouse, chicken and frog (Table S5). Pair-wise comparisons were performed among the three species and significant conservation of gene expression was found when comparing orthologous gene pairs to random gene pairs within each pair-wise comparison (Figure 4). When, within each comparison, the correlation between the gene expressions of an orthologous gene pair was higher than 95% quantile of random gene pairs (as background), we labeled the orthologous gene pair as having a conserved expression pattern. In total, 11.3% (439 genes out of 3,892 genes) chicken-mouse orthologous genes, 10.9% (425 genes) chicken-frog orthologous genes, and 5.01% (195 genes) mouse-frog orthologous genes show a conserved gene expression profile within each pair-wise comparison.

### Homologous tissues are more similar in vertebrates in terms of expression

**Figure 5:**
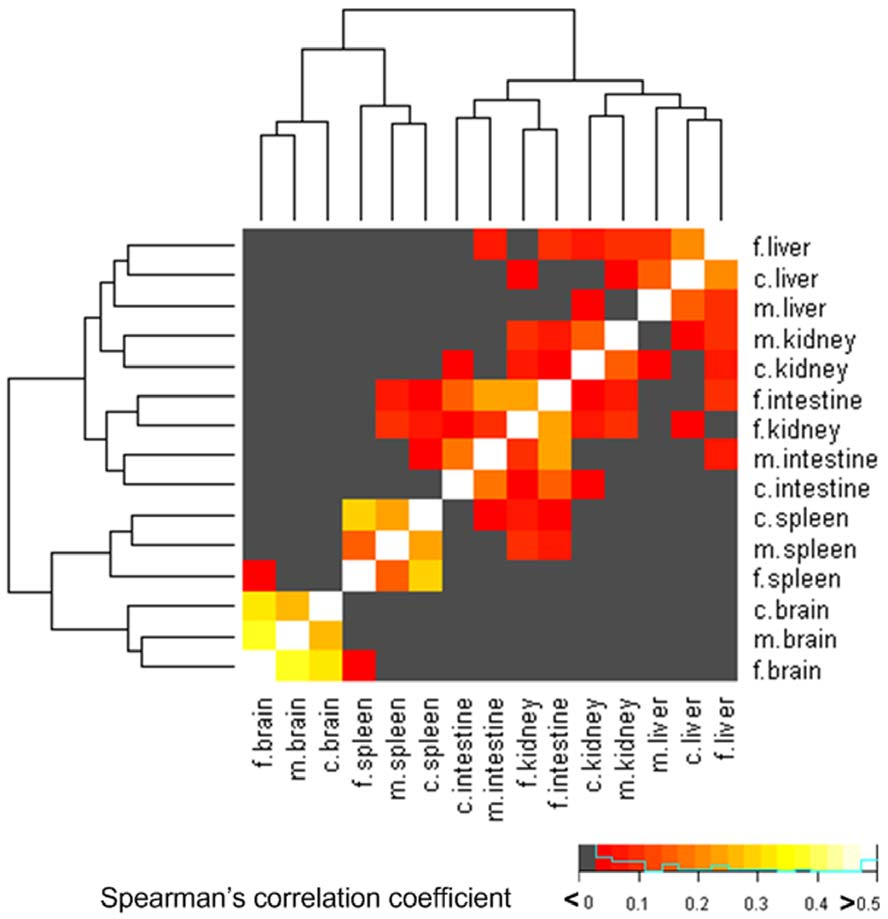
Heat map of correlation coefficients (Spearman) between five common tissues (m: mouse, c: chicken, and f: frog) in three different species. The dendogram shows the clustering of tissue types according to the Spearman's correlation coefficient among tissue types. The color-coded scale of correlation is at bottom right and that the right of scale (lighter colors) signifies higher correlation.

Besides testing conservation of gene expression of orthologous genes between species, we also tested whether homologous tissues (for example, brain tissues in mouse, chicken, and frog) are more similar to each other compared to non-homologous tissues. After transforming gene expression intensities to relative expression ratios (RA) across the same panel of tissues, a comparison between global gene expression profiles among tissues in different species was possible. The rank correlation coefficient among different tissues showed that homologous tissues in three different species are more similar compared to non-homologous tissues (Figure 5); especially brain tissues are highly correlated within the three species indicating evolutionary constraints are posed on brain gene expression profiles. In contrast, kidney and intestine showed a relatively low conservation, this may suggest less evolutionary constraints are posed on organs, e.g. kidney or intestine, with more contact with outside environment comparing to more closed organs, e.g. brain.

## Discussion

### Gene expression distribution in various chicken tissues

The main objective of this study was to survey gene expression profiles across a set of eight normal chicken tissues. We present a microarray expression dataset surveying about 8,792 chicken Ensembl genes across 8 different chicken tissue types in 5-fold (brain, bursa of Fabricius, kidney, liver, lung, small intestine, spleen, and thymus). For most genes the distribution of expression is observed across several different tissues (Figure 1c). For 723 genes, a single-tissue-specific pattern is seen, while 2,476 genes were found to be expressed in all eight tissues. The genes with expression across the eight tissues indicate their universal biological function in cells and therefore can be considered as genes with “housekeeping functions”, although a proper definition of such genes would require a comprehensive sampling of tissues for the whole organism. The GO term enrichment analysis of housekeeping genes show the enriched biological processes GO terms like “translation”, “protein folding”, “protein localization”, “rRNA processing” and “regulation of gene expression” (Table S4). This confirmed that our definition of “housekeeping gene” was valid.

### Housekeeping genes are compact compared to tissue-specific genes and tend to be co-localized in regions of highly-expressed genes

The on average smaller size observed for the housekeeping genes is due to both a shorter coding sequence as well as a shorter intron length. Furthermore, the smaller size of the intergenic region also contributes to a higher gene density of the areas containing the housekeeping genes, suggesting a selection for compactness, which has also been reported in human [7], [8], this might reduce the costs of transcription of housekeeping genes. It has been shown that translation is more costly than transcription [20], and the shorter length of the coding sequences in housekeeping genes is likely the result of selection for economy of translation. On the other hand, the tissue-specific genes are longer, because of their higher number of functional domains and relative more complex protein architecture as was previously reported in human [8]. Likewise, regulation of expression of these genes in a number of specific tissues might have resulted in a large number of cis-regulatory elements and would need larger regulatory “spaces” resulting in larger introns and intergenic regions.

The hypothesis for the existence of RIDGEs is that evolution favors highly expressed genes to be co-localized, as transcription of one gene would help the chromatin of neighboring genes to “open up” during transcription. The favorable distribution of housekeeping genes within RIDGEs again indicates that these genes need to be expressed at relative higher levels (Figure 2) and at a larger number of physiological conditions (“housekeeping functions”).

### Expressions profiles of orthologous genes are conserved in vertebrates

In contrast to direct sequence comparisons of orthologous genes, the comparison of the gene expression profiles of orthologous genes has a number of caveats. First of all, the expression levels of genes are dynamic and change with developmental and physiological state. Secondly, for all downloaded gene expression survey data, the tissue samples surveyed in mouse, chicken and frog [11], [16], [17] are only a part of the whole organs, representing the average of millions of cells of several different types.

Nevertheless, the expression of orthologous genes is generally well conserved as compared to random gene pairs (Figure 4). If gene expression were to evolve in accordance with neutral theory [21], the expression of orthologous genes would be the same as random gene pairs, while our results suggest that gene expression is under some selection constraint during evolution. This is in agreement with previous study comparing human and mouse where high proportion of orthologous genes showed positive correlation [15], our study also confirms the conservation of core gene expression in vertebrates found in previous study [16]. Furthermore, the overall correlation distributions of orthologous gene expressions (see Figure 4) are similar when comparing each pairs among the three species mouse, frog and chicken, this again indicates that the proportions of genes with conserved expression profiles between any two pairs of species are similar, but the mechanism of this conservation is still unknown, and future researches are needed to study the mechanism underneath gene expression conservation.

## Materials and Methods

### Microarray data

The microarray data was downloaded from GEO (http://www.ncbi.nlm.nih.gov/geo/) (accession number: GSE17108), the data was published in a previous study describing a transcriptome map in the chicken genome [11]. In the study, they used the ARK-Genomics G. gallus 20 K oligonucleotide microarray (GEO platform accession: GPL8861) representing most known and predicted chicken genes to investigate global gene expression patterns among 40 tissue samples representing eight adult tissues (brain, bursa of Fabricius, kidney, liver, lung, small intestine, spleen, and thymus) in chicken (5 biological replicates per tissue type), each individual sample was hybridized with a common reference pool (pool of RNA samples from all individual samples). All individual samples were labeled with Cy3, common reference was labeled with Cy5.

### Data processing, normalization, and statistical analysis

We used R/Bioconductor [22] package Limma [23] to analyze the array data. The *.gpr files were imported into R [24] (version 2.8.0), median values of both foreground and background intensities were extracted and used in the analysis. We gave any spot with FLAG-value less than −50 (these spots were flagged as “bad spot” by GenePix (Molecular Devices, Inc.) program or manually) a weight of 0.01, and all the other spots we gave weights of 1. The raw data was normalized in R using variance stabilizing normalization (VSN) methods implemented in package vsn [25]. The normalized intensities of the green channel (representing all individual tissue samples) were used as gene expression data in the analysis and the data points for those spots (both genes and negative controls) with low weight (0.01) were removed in further analysis. The gene expression data was first averaged within each tissue type among the five biological replicates, and then the gene expression data for probes targeting the same Ensembl genes/entrez gene were averaged.

### Gene Ontology term enrichment analysis

All the genes having a chicken Ensembl gene ID were mapped to their 1-to-1 human orthologous genes using bioconductor package biomaRt [26] through the Ensembl Genome Database. The GO term enrichment analysis was subsequently performed using human gene annotation using R package GOstats. A conditional hypergeometric test algorithm provided within GOstats package was applied to GO enrichment analysis. The conditional hypergeometric test identifies a GO term as significant if there is evidence beyond that provided by its significant children. Only the enriched GOBP (Gene Ontology Biological Process) terms with raw p-values<0.01 were used for biological interpretation in this study.

### Comparing 1-1-1 orthologous gene expression conservation

Orthologous genes for mouse (*Mus musculus*), chicken (*Gallus gallus*), and frog (*Xenopus tropicalis*) were downloaded from Ensembl. The normalized gene expression data for mouse and frog were downloaded from the functional landscape of mouse gene expression website (http://hugheslab.med.utoronto.ca/Zhang) and the Conservation of Core Gene Expression in Vertebrate Tissues: Supplementary Data website (http://hugheslab.ccbr.utoronto.ca/supplementary-data/vertebrate_expression), respectively. The expression data of chicken in this study was normalized using the same method as used in these two previous studies [16], [17]. The gene expression data from different species using different species-specific microarray platforms are not directly comparable. To enable cross-species gene expression comparisons, we used relative mRNA abundance (RA) among tissues introduced by Liao and Zhang [15]. Gene expression levels were calculated as ratios between the expression intensity of gene X in one particular tissue divided by sum of expression intensities of gene X in all tissues included in the analysis.

## Supporting Information

Table S1 Expression data of 8908 genes in eight chicken tissues.
(1.82 MB XLS)

Table S2 Lists of tissue specific genes and housekeeping genes.
(0.20 MB XLS)

Table S3 GO enrichment results of tissue-specific genes.
(0.02 MB XLS)

Table S4 GO enrichment result of housekeeping genes.
(0.02 MB XLS)

Table S5 List of 3,892 1∶1∶1 orthologous genes in mouse, chicken and frog downloaded from Ensembl database via biomaRt package.
(1.15 MB XLS)
